# *AINTEGUMENTA* and the D-type cyclin *CYCD3;1* regulate root secondary growth and respond to cytokinins

**DOI:** 10.1242/bio.013128

**Published:** 2015-09-04

**Authors:** Ricardo S. Randall, Shunsuke Miyashima, Tiina Blomster, Jing Zhang, Annakaisa Elo, Anna Karlberg, Juha Immanen, Kaisa Nieminen, Ji-Young Lee, Tatsuo Kakimoto, Karolina Blajecka, Charles W. Melnyk, Annette Alcasabas, Celine Forzani, Miho Matsumoto-Kitano, Ari Pekka Mähönen, Rishikesh Bhalerao, Walter Dewitte, Ykä Helariutta, James A. H. Murray

**Affiliations:** 1 Department of Molecular Biosciences, Cardiff School of Biosciences, Cardiff University, Cardiff, Wales CF10 3AX, UK; 2 Department of Biological Sciences, Osaka University, Graduate School of Science, 1-1 Machikaneyama-cho, Toyonaka, Osaka 560-0043, Japan; 3 Department of Biosciences, Institute of Biotechnology, Viikinkaari 1 (P.O.Box 65), 00014, University of Helsinki, Helsinki, Finland; 4 Department of Plant Physiology, Umeå University, Umeå SE-901 87, Sweden; 5 School of Biological Sciences, College of Natural Science, Seoul National University, 1 Gwanak-ro, Gwanak-gu, Seoul 08826, Korea; 6 Sainsbury Laboratory, Cambridge University, Bateman Street, Cambridge CB2 1LR, UK

**Keywords:** Cytokinins, Secondary growth, Cyclin D, AINTEGUMENTA, Root development

## Abstract

Higher plant vasculature is characterized by two distinct developmental phases. Initially, a well-defined radial primary pattern is established. In eudicots, this is followed by secondary growth, which involves development of the cambium and is required for efficient water and nutrient transport and wood formation. Regulation of secondary growth involves several phytohormones, and cytokinins have been implicated as key players, particularly in the activation of cell proliferation, but the molecular mechanisms mediating this hormonal control remain unknown. Here we show that the genes encoding the transcription factor AINTEGUMENTA (ANT) and the D-type cyclin CYCD3;1 are expressed in the vascular cambium of Arabidopsis roots, respond to cytokinins and are both required for proper root secondary thickening. Cytokinin regulation of *ANT* and *CYCD3* also occurs during secondary thickening of poplar stems, suggesting this represents a conserved regulatory mechanism.

## INTRODUCTION

The phytohormone cytokinin regulates several root developmental processes, including elongation, apical meristem maintenance and vascular morphogenesis (Ferreira and Kieber, 2005; Higuchi et al., 2004; Mahonen et al., 2000; Nishimura et al., 2004). Cytokinin signalling, together with other hormonal cues (Miyashima et al., 2013), is also important for root secondary growth, which involves thickening of the root via proliferation of cambial cells (Spicer and Groover, 2010; Nieminen et al., 2004). Arabidopsis mutants defective in cytokinin biosynthesis develop thinner roots with a cambium composed of fewer cells, a phenotype rescued by exogenous cytokinin application (Matsumoto-Kitano et al., 2008). Cytokinin therefore appears to act at least in part through promoting cell division and hence activation of the mitotic cell cycle.

*CYCD* genes encode conserved regulatory sub-units of cyclin D-cyclin-dependent kinase (CYCD-CDK) complexes that promote cell cycle progression in animals and plants (Menges et al., 2007; Morgan, 1997). The CYCD3 subgroup of CYCDs is conserved across all higher plants (Menges et al., 2007) and has three members in Arabidopsis: CYCD3;1, CYCD3;2 and CYCD3;3. CYCD3s control progression through the G1/S transition (Menges et al., 2006), and also regulate the length of the temporal period of mitotic cell division during aerial organ development (Dewitte et al., 2007).

*CYCD3* genes are induced by cytokinins (Menges et al., 2007, 2006; Riou-Khamlichi et al., 1999) and are rate-limiting for cytokinin responses in Arabidopsis shoots (Dewitte et al., 2007; Riou-Khamlichi et al., 1999). Here, we identify novel roles for CYCD3;1 and the AINTEGUMENTA transcription factor in root secondary growth. Prolonged expression of *CYCD3;1* in leaves caused by ectopic expression of *ANT* (Mizukami and Fischer, 2000) has led to the suggestion that *CYCD3;1* is a target of ANT (Anastasiou and Lenhard, 2007; Wu et al., 2011). However, we show here that CYCD3 and AINTEGUMENTA play independent roles in regulating secondary thickening in roots, but provide evidence that they are co-regulated by cytokinins.

## RESULTS AND DISCUSSION

### *CYCD3;1* is rate-limiting for root secondary thickening

**Fig. 1.**
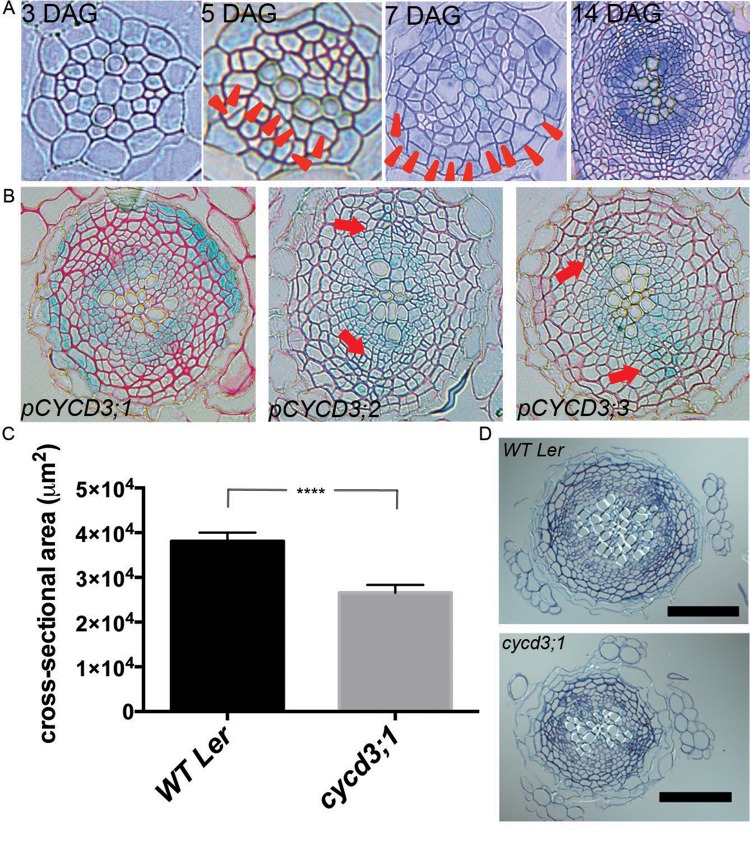
***CYCD3;1* is expressed in the root cambium and regulates secondary growth.** (A) Transverse sections of roots taken immediately below the hypocotyl at time points shown. Arrowheads indicate recently formed cell walls. (B) Expression patterns of *CYCD3 promoter:GUS* reporters. *pCYCD3;1* drives GUS expression in the cambium and the pericycle (left panel), whereas *pCYCD3;2* and *pCYCD3;3* drive GUS expression in the cambium and phloem (middle and right panel; red arrows). (C) Stele cross-sectional area in 17 DAG L*er* and *cycd3;1* roots. ****P<0.0001; Error bars represent s.e.m. (D) Transverse sections of roots in C. Scale bars: 100 µm.

Secondary growth involves cell proliferation in the cambium (Fig. 1A). Given the known requirement for cytokinin signalling for root secondary thickening, and involvement of CYCD3s in shoot growth responses to cytokinins, we analysed the expression patterns of *CYCD3s* using promoter*:GUS* constructs. *pCYCD3;1:GUS* expression was observed in the innermost and outermost regions of the stele of roots undergoing secondary growth (Fig. 1A,B). These regions contain the cambium and pericycle cells respectively, both of which contribute to secondary thickening. *pCYCD3;2:GUS* and *pCYCD3;3:GUS* expression was detected in the cambium and in the phloem cells perpendicular to the primary xylem axis. *CYCD3;1* expression has also been reported in whole mounts of root tissue undergoing secondary growth (Collins et al., 2015) and the vascular tissue of vegetative and flowering Arabidopsis shoot apices (Dewitte et al., 2003). Furthermore, expression of *CYCD3* genes in the vascular tissue of roots undergoing primary growth was recently inferred (Collins et al., 2015) from microarray data obtained from fluorescently activated cell-sorted primary root cells (Brady et al., 2007). Collins et al. (2015) also analysed expression of *CYCD3* genes in shoot cambium development with the same transcriptional fusion reporter lines used here and detected expression of all three *CYCD3* genes in this tissue. These data suggest that *CYCD3* genes are expressed in radially proliferating tissues and play an active role in secondary growth.

*CYCD3* genes were recently shown to contribute to secondary growth in Arabidopsis stems with reduced hypocotyl diameter and vascular cell number in the *cycd3;1-3* triple mutant generated in the Columbia background (Collins et al., 2015), although the contribution of individual *CYCD3* genes was not determined. This supports a scenario in which *CYCD3s* are core regulators of cambial cell proliferation in both shoots and roots. Indeed, our expression data suggest that *CYCD3s* could play a role in root secondary growth. We compared the stele cross-sectional area in *cycd3;1* to that of WT immediately under the hypocotyl during secondary growth (Fig. 1A). In order to avoid confounding effects from other polymorphisms in the L*er* and Col-O backgrounds, we analysed the *cycd3;1* allele in the L*er* background, in which it was initially generated (Parinov et al., 1999). At 17 days after germination (DAG), *cycd3;1* roots displayed a narrower stele than WT counterparts (Fig. 1C). Concomitant with reduced cell division activity, *cycd3;1* roots had a reduced number of vascular cells (supplementary material Fig. S1). We conclude that CYCD3;1 promotes root secondary growth. Root diameter was reduced to a similar extent in both *cycd3;1* and *cycd3;1-3* triple mutants in the L*er* background (supplementary material Fig. S2A,B), consistent with *CYCD3;1* being primarily required among the *CYCD3s* genes for secondary growth in L*er* roots.

### ANT contributes to root secondary thickening

To identify potential regulators of *CYCD3;1* expression, we performed genome-wide Pearson's correlation tests for *CYCD3;1* coexpression with all known transcription factors across >500 public Affymetrix ATH1 microarray datasets annotated as conducted on root tissues. Expression of *CYCD3;1* was most highly correlated with *AINTEGUMENTA* (*ANT*) (supplementary material Table S2). ANT is a member of the AP2 (APETALA2)/EREBP (Ethylene Response Element Binding Protein) family, and falls into the ANT-lineage of the AP2-like subgroup (Kim et al., 2006). Several members of the AP2-like subgroup are associated with developmental regulation during growth of young tissues (Nole-Wilson et al., 2005). ANT is involved in the control of lateral aerial organ size via the regulation of cell proliferation (Mizukami and Fischer, 2000), and is associated with fruit growth in apple (Dash and Malladi, 2012) and seasonal bud dormancy in hybrid aspen (Karlberg et al., 2011). Moreover, ectopic expression of ANT in leaves increased levels of *CYCD3;1* mRNA (Mizukami and Fischer, 2000). Therefore, ANT is a candidate regulator of *CYCD3;1* during secondary root growth.

**Fig. 2.**
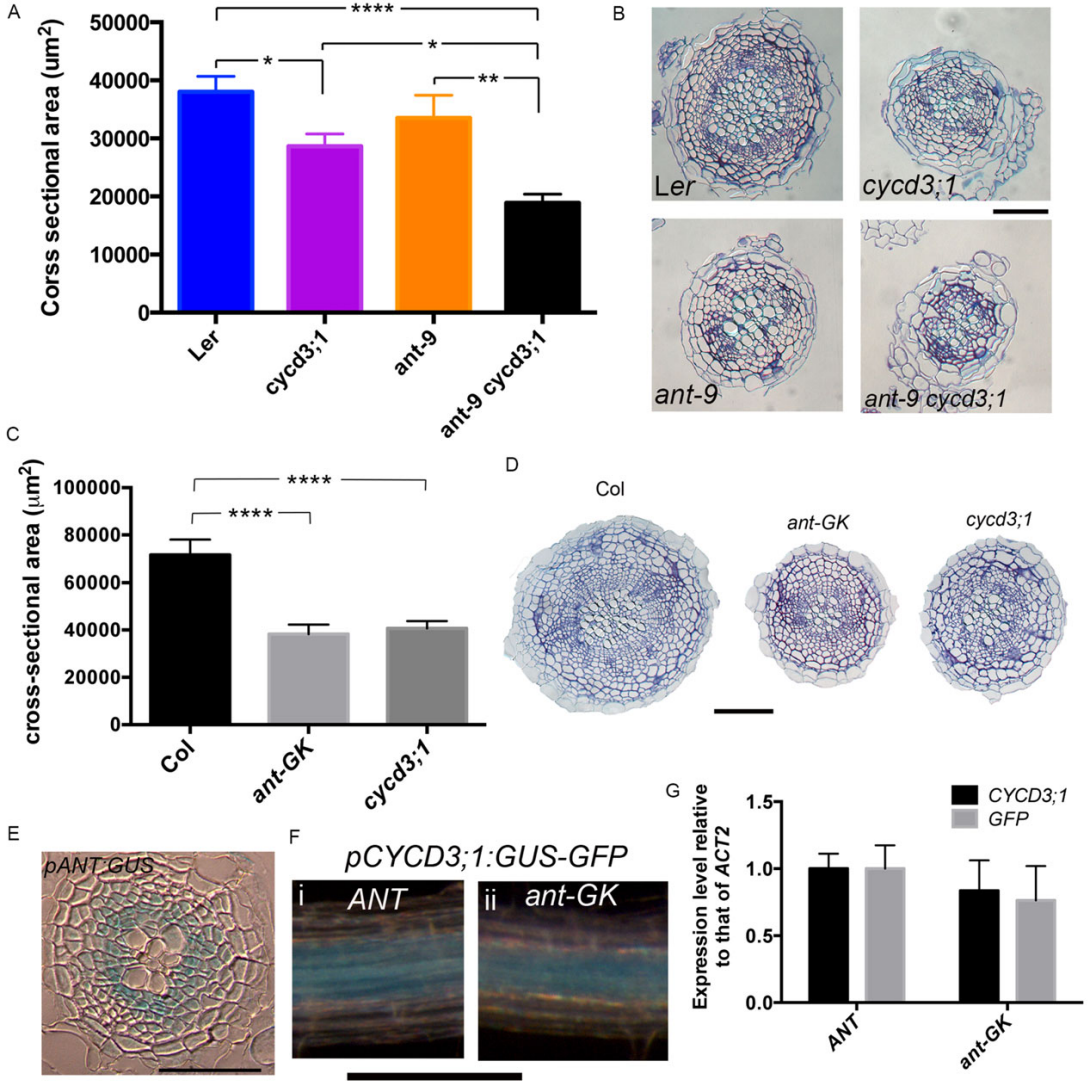
**Interaction between *ANT* and *CYCD3;1* in root secondary growth.** (A,C) Stele cross-sectional area in 30 DAG L*er, cycd3;1*, *ant-9* and *ant-9 cycd3;1* roots (A) and 21 DAG *Col-0, ant-GK* and *cycd3;1 (Col-0)* roots (C). *0.01<*P*<0.05, **0.001<*P*<0.01, ****P<0.0001; Error bars represent s.e.m. (B,D) Transverse sections of roots in A (B) and C (D). Scale bars: 100 µm. (E) Transverse section from a GUS-stained 14 DAG *pANT:GUS* root. Scale bar: 50 μm. (F) *pCYCD3;1:GUS-GFP* expression in 14 DAG *ANT* (i) and *ant-GK* (ii) roots. Scale bar: 200 µm. (G) qPCR analysis of *CYCD3;1* and *GFP* in 16 day-old *ANT pCYCD3;1:GUS-GFP* and *ant-GK pCYCD3;1:GUS-GFP* roots. Error bars: s.d. in four biological replicates, each from eight pooled roots.

To test this hypothesis, the genetic interaction between *ANT* and *CYCD3;1* was investigated. The original *ant-9* allele isolated in the Landsberg *erecta* ecotype was investigated. No statistically significant reduction in root cross-sectional area was observed in *ant-9* mutants (Fig. 2A,B). However, cross-sectional area was reduced to a greater extent in *ant-9 cycd3;1* double mutants than in the *cycd3;1* single mutant (Fig. 2A,B), suggesting a contribution of ANT to secondary thickening through a synergistic genetic interaction between *ANT* and *CYCD3;1*. To further investigate the contribution of ANT to secondary growth, we identified a new *ant* mutant in the *Col-0* background derived from the GABI-Kat collection (supplementary material Fig. S3A). Homozygous *ant-GK* plants display the characteristic *ant* mutant phenotypes of reduced floral organ size (supplementary material Fig. S3A) and fail to produce seeds. Interestingly, root cross-sectional area and vascular cell number were reduced in *ant-GK* mutants to a similar extent as the reductions seen in *cycd3;1* mutants (Fig. 2C,D and supplementary material Fig. S1).

To confirm that effects of loss of functional ANT and CYCD3;1 in the shoot did not influence or cause the root phenotypes described here, we grafted WT shoot scion onto *ant-GK* and *cycd3;1* root scion. The phenotypes remained (supplementary material Fig. S4), confirming that these are independent root phenotypes. Furthermore, root elongation was not affected by *ant* and *cycd3;1* mutations (supplementary material Fig. S6), demonstrating that the secondary growth phenotype was not caused by altered root ontogeny dynamics. The *ant-GK* allele is in the *Col-0* background, whereas the *ant-9* allele is in *Ler*, possibly explaining the difference in the severities of these phenotypes. We conclude that ANT also regulates root secondary thickening.

Relatively high levels of *ANT* mRNA have been reported in roots (Elliott et al., 1996), but the root expression pattern remains unknown. Expression of a *pANT:GUS* reporter was readily detected in the root cambium (Fig. 2E). Supporting this, in a transgenic line expressing a histone H2B-YFP fusion under the control of the *ANT* promoter (*pANT:H2B-YFP*), relatively strong fluorescence was observed in the stele of the more mature root (supplementary material Fig. S6).

Regulation of *CYCD3;1* by ANT in shoot organs has been proposed but not demonstrated (Anastasiou and Lenhard, 2007; Horiguchi et al., 2009; Schruff et al., 2006). To test whether ANT might regulate *CYCD3;1* expression in the root, a *pCYCD3;1:GUS-GFP* reporter was introduced into the *ant-GK* mutant. Visual comparison of *pCYCD3;1:GUS-GFP* expression in sibling F3 *ant-GK* and WT plants did not reveal a reduction in expression in *ant-GK* homozygous roots (Fig. 2F). However, qPCR analyses of root mRNA revealed a small reduction of both native *CYCD3;1* and *GUS-GFP* transcript abundance in *ant-GK pCYCD3;1:GUS-GFP* mutants (Fig. 2G). This could however be explained by a relative decrease in the abundance of *CYCD3;1*-expressing cambial cells in the *ant-GK* mutant. Therefore, whilst it remains possible that ANT regulates *CYCD3;1*, taken together with the genetic evidence no strong regulation is indicated. Supporting this conclusion, *CYCD3;1* transcript abundance was not reduced in *ant-9* roots (supplementary material Fig. S7). Furthermore, an additive phenotype in the *ant-9 cycd3;1* double mutant was also shown for Arabidopsis petal epidermal cell size, and *CYCD3;1* expression was not reduced in young *ant-9* flowers (Randall et al., 2015).

### Cytokinin signalling regulates both *ANT* and *CYCD3;1* expression

*ant, cycd3;1* and cytokinin synthesis/response mutants show common phenotypes of defective root secondary thickening (Hejatko et al., 2009; Matsumoto-Kitano et al., 2008). Since cytokinins are known to regulate *CYCD3;1* activity in shoot tissues (Dewitte et al., 2007), we assessed cytokinins as potential regulators of *ANT* and *CYCD3;1* in the root cambium.

We first analysed their expression in response to exogenous cytokinin. qRT-PCR analysis of root mRNA showed that following cytokinin application to plants 14 DAG, *ANT* transcript levels were increased 6-fold relative to untreated plants, correlating with a smaller increase in *CYCD3;1* transcript levels (supplementary material Fig. S8A). Supporting induction of *CYCD3;1* by cytokinins, *pCYCD3;1:GUS* expression was also induced in roots after addition of the synthetic cytokinin kinetin (supplementary material Fig. S8B).

**Fig. 3.**
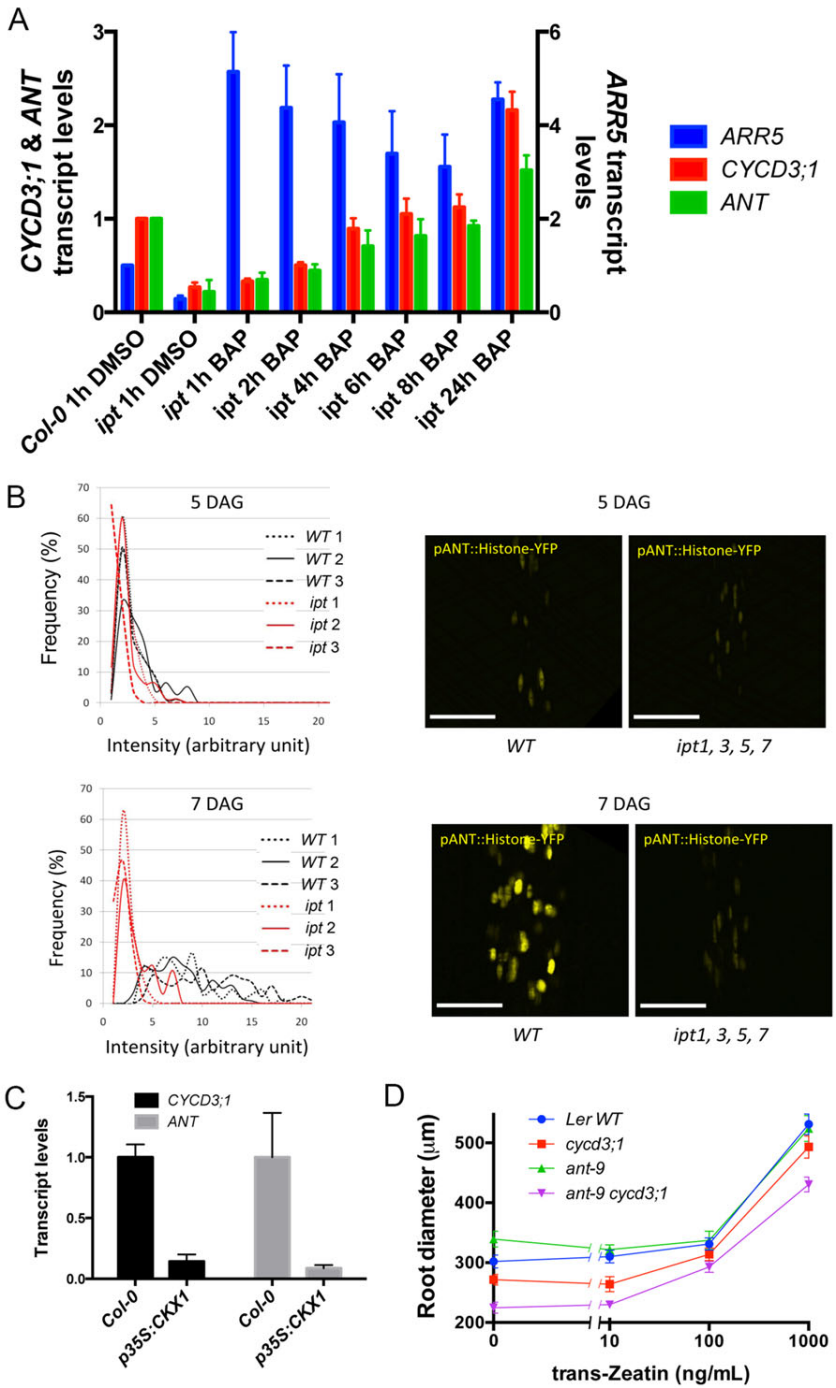
***ANT* and *CYCD3;1* respond to cytokinins in root secondary thickening.** (A) qPCR analysis of *ARR5, ANT* and *CYCD3;1* in *Col-0* and *ipt1;3;5;7* roots treated with DMSO and *ipt1;3;5;7* roots treated with 1 µM BAP for the periods indicated. Error bars represent s.d. from 3 biological replicates. (B) Intensity of YFP signal in lines expressing *pANT:H2B-YFP* at 5 DAG and 7 DAG in *WT* plants vs *ipt1,3,5,7* plants. *WT1-3* and *ipt1-3* each represent three independent T3 lines homozygous for *pANT:H2B-YFP*. *ipt1-3* are also homozygous for *ipt1;3;5;7* alleles. In each line, signal intensity was measured from >50 nuclei. Frequency distributions of signal intensity are shown. (C) Transcript levels of *CYCD3;1* and *ANT* in *Col-0* and *p35S:CKX1* roots. s.d. in three biological replicates is shown. (D) Diameter of L*er, cycd3;1, ant-9* and *ant-9 cycd3;1* roots following tZ treatments. Roots were grown for 11 days then transferred to media containing (or not for control) tZ. Error bars represent s.e.m.

To analyse induction of *ANT* and *CYCD3;1* with greater resolution, we measured expression at several time points following addition of cytokinins to *ipt1,3,5,7* mutants. These mutants carry loss-of-function alleles for four *ipt* genes (*ipt1,3,5,7*), which encode isopentenyl transferases involved in cytokinin biosynthesis. These multiple mutants have reduced levels of isopentenyladenine as well as trans-zeatin (tZ), a cytokinin shown to have an effect on cambium proliferation (Matsumoto-Kitano et al., 2008; Miyawaki et al., 2006). Since these mutants fail to undergo proper secondary growth in roots (Matsumoto-Kitano et al., 2008), the use of this mutant should limit the amount of background *ANT* and *CYCD3;1* expression. Consistent with cambium-expression of *ANT* and *CYCD3;1*, expression of these genes was reduced in the *ipt1,3,5,7* mutant (Fig. 3A). Elevated *ANT* and *CYCD3;1* expression was observed from 4 h after incubation with BAP (Student's *t*-test, *P*<0.05; *n*=3 in each case), increasing until 24 h.

We then investigated the cytokinin requirement for the increased ANT promoter activity observed during the transition from primary to secondary root growth (Fig. 3B). We analysed expression of *pANT:H2B-YFP* in *ipt1,3,5,7* mutants. During the activation stage around 5 DAG (Fig. 1A), when the first cell division event in the procambium occurs, weak YFP signal from the *pANT:H2B-YFP* construct was detected in the roots of both WT and *ipt1;3;5;7* plants (Fig. 3B top). Quantitation of the YFP signal from confocal images showed similar signal intensity in both WT and *ipt* mutant plants (Fig. 3B left). However, whereas in WT plants the signal intensified in roots during the transition stage (7 DAG), when procambial cells begin to proliferate, in *ipt1;3;5;7* roots it did not (Fig. 3B). We suggest that the increase of *ANT* expression observed in WT roots depends on normal levels of cytokinins. Alternatively, *ANT* expression might be delayed due to a delay in vascular tissue development in the *ipt1;3;5;7* mutant.

To further investigate the dependence of *ANT* and *CYCD3;1* expression on cytokinin, we used *p35S:CKX1* plants overexpressing cytokinin oxidase leading to lower levels of cytokinins (Werner et al., 2001). These displayed reduced abundance of *ANT* and *CYCD3;1* transcripts in roots (Fig. 3C). Taken together, these results strongly indicate regulation of *ANT* and *CYCD3;1* in the root vascular tissue by cytokinins.

### ANT and CYCD3;1 are involved in the regulation of root secondary growth by cytokinins

To determine whether ANT and CYCD3;1 are part of the signalling mechanism by which cytokinins promote secondary thickening in roots, the root thickening response to cytokinins was analysed in mutants. Initially, the response of *cycd3;1* roots to cytokinins was compared to WT. Although *cycd3;1* roots were thinner than WT and remained so with low concentrations of tZ, higher concentrations of tZ restored secondary thickening to a level comparable with WT (supplementary material Fig. S9). We next compared this response in WT, *ant-9, cycd3;1*, and *ant-9 cycd3;1* double mutants. As previously observed (Fig. 2A and supplementary material Fig. S7), without addition of cytokinins *cycd3;1* and *ant-9 cycd3;1* roots showed reduced diameter compared to WT L*er* roots, the double mutant being thinner than the single mutant (Fig. 3D). All genotypes respond to tZ by increasing in diameter but notably the *ant-9 cycd3;1* double mutant remains thinner than other genotypes at higher tZ concentrations (1000 ng/µl; Fig. 3D), supporting a synergistic interaction between *ANT* and *CYCD3;1* and implying that these factors independently contribute to radial cell division activity in the cambium. This data also indicates that other factors can mediate the response of the cambium to cytokinins in the absence of ANT and CYCD3;1.

### Conserved roles for *ANT* and *CYCD3;1* in secondary growth

**Fig. 4.**
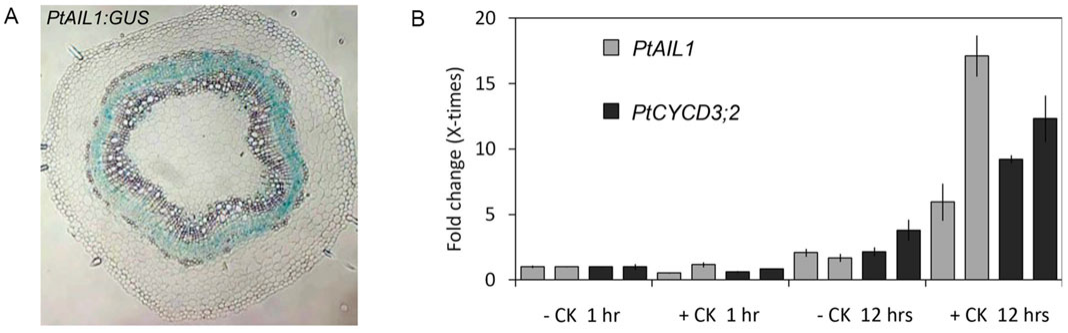
**Poplar ANT is expressed in the cambium and is induced by cytokinins.** Cross-section of a *Poplar pAIL1:GUS* stem following GUS assay (A) and qPCR analyses of *PtANT* and *PtCYCD3;2* transcripts following treatments of cytokinin (100 nM 2iP) or mock treatments for one or twelve hours (B). Two individuals represented separately in adjacent bars were used for the experiment. Transcript levels were normalized to *PttTUA2.* Error bars: s.d. in four technical replicates.

Both *ANT* and *CYCD3* genes are widely conserved amongst angiosperms (Kim et al., 2006; Menges et al., 2007) and expression of the poplar *ANT* orthologue has been reported in cambial tissue (Schrader et al., 2004; Zhang et al., 2011). It was also reported that down-regulation of *ANT* and *CYCD3* orthologues in hybrid aspen *(P. tremula x tremuloides)* is required for bud-growth cessation in this species (Karlberg et al., 2011). We analysed the activity of the poplar *ANT* orthologue *AIL1* in stems undergoing secondary growth, and observed activity within the cambium (Fig. 4A), consistent with a potential role for *AIL1* in the regulation of secondary growth in this species. The activity of a promoter sequence designated *PttANT* was also recently reported in the cambium (Etchells et al., 2015); this promoter sequence was derived independently but appears to be that of *PttAIL1* according to the locus identity, supporting the expression pattern described here. As cytokinins also regulate secondary growth in poplar (Nieminen et al., 2008), we analysed the expression of *PtAIL1* and the poplar *CYCD3* homologue *PtCYCD3;2* following cytokinin treatments. It should be noted that the *CYCD3* gene number suffixes denote arbitrary order of naming in that species (Renaudin et al., 1996). qRT-PCR analyses revealed increases in relative abundances of both *PtAIL1* and *PtCYCD3;2* transcripts after twelve hours of cytokinin treatment (Fig. 4B). Therefore, we propose that the regulation of secondary growth by cytokinin-induced *ANT* and *CYCD3;1* is conserved in higher plants.

### Conclusions

The transcription factor ANT and the cyclin CYCD3;1 play independent roles in regulating cell division during secondary growth of Arabidopsis roots. *cycd3;1*, *ant* and cytokinin biosynthesis and receptor mutants share a common failure in cambial proliferation, and we show that these components act in conserved pathways linking cytokinins to developmentally regulated cell proliferation.

## MATERIALS AND METHODS

### Plant material and growth

Arabidopsis plants were grown in 16 h days at 22°C on MS medium containing 1.5% sucrose, 0.5 g/l 2-(N-morpholino)ethanesulfonic acid and 1% agar. *CYCD3 promoter:GUS* reporter lines, *cycd3;1, ant-9, ipt1;3;5;7* and *cre2;3;4* mutants have been described (Dewitte et al., 2007; Elliott et al., 1996; Higuchi et al., 2004; Miyawaki et al., 2006; Mizukami and Fischer, 2000). The *cycd3;1* mutant initially isolated from a Landsberg *erecta* (L*er*) DS element insertion library (Parinov et al., 1999) was backcrossed twice to L*er* WT, and the triple *cycd3;1-3* mutant in the L*er* background was generated by crossing *cycd3;1* with *cycd3;2,* and backcrossing lines hemizygous for both alleles to L*er* twice before introgressing the *cycd3;3* allele from the EXOTIC collection (Dewitte et al., 2007). The *ant-GK* mutant (GK-874H08; TAIR accession no.: 1006453905) is described in supplementary material Fig. S3. The *pCYCD3;1:GUS-GFP* and *pANT:Histone2b-YFP* (*pANT:H2B-YFP)* reporters were constructed in pKGWFS7 (Karimi et al., 2002). 947 bp of DNA upstream (representing sequence to the adjacent gene) of the *CYCD3;1* start codon was used. 5137 bp of *ANT* upstream sequence was used for *pANT:H2B-YFP*, and 6291 bp for *pANT:GUS* which was isolated from a lambda EMBL3 library (David Smyth and Brian Kwan, Monash University, Melbourne, Australia). Scion of *Atipt1;3;5;7* was grafted onto WT stock and used for floral dipping (Clough and Bent, 1998).

WT *Populus tremula x tremuloides* line T89 was used for qPCR analyses. *P. tremula x tremuloides* seedlings were grown for one month in long-day greenhouse conditions at 22°C in 5 litre pots. The *pAIL1:GUS* line has been described (Karlberg et al., 2011).

### Arabidopsis micrografting

Arabidopsis plants were grafted according to a published protocol (Turnbull et al., 2002) with the following modifications. Ethanol sterilised Arabidopsis seeds were germinated on 1/2 Murashige and Skoog (MS) medium plus 1% Difco agar (pH 5.7; 1% sucrose) and grown on vertically-mounted Petri dishes under long (16 h of 80–100 µmoles light, *Col-0* and *ant-GK* grafts) or short day conditions (8 h of 80–100 µmoles light, *Ler* and *cycd3;1* grafts) at 20°C. Grafting was performed under sterile conditions in a laminar flow hood with a Zeiss portable dissecting microscope. 5–6 day-old seedlings were transferred to 9 cm Petri dishes that contained one layer of 2.5×4 cm sterilised Hybond N membrane (GE Healthcare) on top of two sterilised 8 cm disks of 3 mm Chr Whatman paper (Scientific Laboratory Supplies). The Whatman paper and Hybond N membrane were kept moist using a 1% sucrose solution (*Col-0* and *ant-GK* grafts) or sterile distilled water (*Ler* and *cycd3;1* grafts). A transverse cut was made through the hypocotyl close to the shoot using a vascular dissecting knife (Ultra Fine Micro Knife; Fine Science Tools). In addition, one cotyledon was removed to assist in aligning the grafted pieces. In the case of self-grafts, an additional 1 mm segment was cut from the hypocotyl and discarded. Grafts were assembled by butt alignment of the two cut halves with no supporting collar. After grafting, Petri dishes were sealed with parafilm, mounted vertically under long (*Col*-0 and *ant-GK* grafts) or short day conditions (*Ler* and *cycd3;1* grafts) and monitored for 7 days at 20°C. Afterwards, successful grafts and un-grafted controls were transferred to 1/2 MS plates with 1% sucrose under long day conditions at 20°C for additional 2–3 weeks. For *ant-GK* related grafts and un-grafted plants, DNA was extracted and genotyping PCR was performed to confirm the genotype of each scion and stock. Roots from stock were sampled and embedded with Leica resin. 5 µm-thin plastic sections were cut at 5 mm below the hypocotyl-root junction and stained with Toluidine Blue O.

### Microscopy and anatomical analyses

Transverse sectioning and GUS assays were described (Mahonen et al., 2006, 2000; Nieuwland et al., 2009). Sections were taken within 1 cm of the root-hypocotyl junction. Microscopy was performed using a Zeiss LSM 710 or a Leica SP5. Roots were stained with 100 μg/ml propidium iodide.

### Cytokinin induction and quantitative PCR

BAP, kinetin and *trans*-zeatin were dissolved in dimethyl sulfoxide and diluted in sterile water. 2iP was dissolved in 20 mM NaPi buffer. Arabidopsis inductions were performed by transplanting onto plates containing cytokinins or equivalent control buffer. Inductions in *P. tremula x tremuloides* seedlings were performed by submerging stem pieces in solutions indicated. *Col-0* and *ipt1,3,5,7* mutants were grown on a nylon mesh (SEFAR NITEX 03-100/44) on vertical plates containing 0.8% Plant Agar (Duchefa), 1% sucrose (Duchefa), 0.5× MS medium including vitamins (Duchefa) and pH adjusted to 5.7–5.8 with 20× MES buffer (Mes monohydrate, Duchefa). 7-day-old plants were transferred with the mesh to plates containing 1 µM BAP or the respective DMSO control. Primary root samples consisting of 40 plants were harvested at 1, 2, 4, 6, 8 and 24 h after the transfer by cutting a few millimeters below the hypocotyl, primary root tips and lateral roots were discarded. RNA was isolated with Qiagen RNeasy Plant Mini Kit with an on-column DNase treatment. Additional DNase treatment with DNase I (RNase-free) (Thermo Scientific) was performed to 1 µg of RNA prior to the oligo-dT-primed cDNA synthesis with First Strand cDNA Synthesis Kit (Thermo Scientific). Expression of *ANT*, *CYCD3;1* and *ARR5* was quantified with primers listed in supplementary material Table S3with HOT FIREPol^®^ EvaGreen^®^ qPCR Mix Plus (no ROX) (Solis Biodyne). The Bio-Rad CFX384 was used with one cycle 95°C for 15 min, 40 cycles each consisting of 95°C for 15 s, 60°C for 30 s and 72°C for 30 s, one cycle 95°C for 10 s followed by melt curve analysis. Raw data values were normalized to the geometric mean of four control genes (Vandesompele et al., 2002) and fold changes calculated in comparison to the *Col-0* expression level. The experiment was performed in triplicate.

For other experiments, RNA was isolated using TriPure (Roche Diagnostics) and cDNA was synthesized using the Ambion Retroscript kit. qRT-PCR analyses were performed as described for Arabidopsis (Menges and Murray, 2002) and *P. tremula x tremuloides* (Nieminen et al., 2008)*.* Gene-specific primers are listed (supplementary material Table S1). Relative transcript levels were quantified using the ΔΔCT method (Livak and Schmittgen, 2001).

### Statistics

Student's *t*-tests were performed in GraphPad Prism. *0.01<*P*<0.05 **0.001<*P*<0.01 ***0.0001<*P*<0.001 ****P<0.0001. Pearson's correlation tests were carried out in R (r-project.org).

## References

[BIO013128C1] Anastasiou E. and Lenhard M. (2007). Growing up to one's standard. *Curr. Opin. Plant Biol.* 10, 63-69. 10.1016/j.pbi.2006.11.002 17134936

[BIO013128C2] Brady S. M., Orlando D. A., Lee J.-Y., Wang J. Y., Koch J., Dinneny J. R., Mace D., Ohler U. and Benfey P. N. (2007). A high-resolution root spatiotemporal map reveals dominant expression patterns. *Science* 318, 801-806. 10.1126/science.1146265 17975066

[BIO013128C3] Clough S. J. and Bent A. F. (1998). Floral dip: a simplified method for Agrobacterium-mediated transformation of Arabidopsis thaliana. *Plant J.* 16, 735-743. 10.1046/j.1365-313x.1998.00343.x 10069079

[BIO013128C4] Collins C., Maruthi, N. M. and Jahn C. E. (2015). CYCD3 D-type cyclins regulate cambial cell proliferation and secondary growth in Arabidopsis. *J. Exp. Bot.* 66, 4595-4606. 10.1093/jxb/erv218 26022252 PMC4507761

[BIO013128C5] Dash M. and Malladi A. (2012). The AINTEGUMENTA genes, MdANT1 and MdANT2, are associated with the regulation of cell production during fruit growth in apple (Malus×domestica Borkh.). *BMC Plant Biol.* 12, 98 10.1186/1471-2229-12-98 22731507 PMC3408378

[BIO013128C6] Dewitte W., Riou-Khamlichi C., Scofield S., Healy J. M. S., Jacqmard A., Kilby N. J. and Murray J. A. H. (2003). Altered cell cycle distribution, hyperplasia, and inhibited differentiation in Arabidopsis caused by the D-type cyclin CYCD3. *Plant Cell* 15, 79-92. 10.1105/tpc.004838 12509523 PMC143452

[BIO013128C7] Dewitte W., Scofield S., Alcasabas A. A., Maughan S. C., Menges M., Braun N., Collins C., Nieuwland J., Prinsen E., Sundaresan V. et al. (2007). Arabidopsis CYCD3 D-type cyclins link cell proliferation and endocycles and are rate-limiting for cytokinin responses. *Proc. Natl. Acad. Sci. USA* 104, 14537-14542. 10.1073/pnas.0704166104 17726100 PMC1964848

[BIO013128C8] Elliott R. C., Betzner A. S., Huttner E., Oakes M. P., Tucker W. Q., Gerentes D., Perez P. and Smyth D. R. (1996). AINTEGUMENTA, an APETALA2-like gene of Arabidopsis with pleiotropic roles in ovule development and floral organ growth. *Plant Cell* 8, 155-168. 10.1105/tpc.8.2.155 8742707 PMC161088

[BIO013128C9] Etchells J. P., Mishra L. S., Kumar M., Campbell L. and Turner S. R. (2015). Wood formation in trees is increased by manipulating PXY-regulated cell division. *Curr. Biol.* 25, 1050-1055. 10.1016/j.cub.2015.02.023 25866390 PMC4406943

[BIO013128C10] Ferreira F. J. and Kieber J. J. (2005). Cytokinin signaling. *Curr. Opin. Plant Biol.* 8, 518-525. 10.1016/j.pbi.2005.07.013 16054432

[BIO013128C11] Hejatko J., Ryu H., Kim G.-T., Dobesova R., Choi S., Choi S. M., Soucek P., Horak J., Pekarova B., Palme K. et al. (2009). The histidine kinases CYTOKININ-INDEPENDENT1 and ARABIDOPSIS HISTIDINE KINASE2 and 3 regulate vascular tissue development in Arabidopsis shoots. *Plant Cell* 21, 2008-2021. 10.1105/tpc.109.066696 19622803 PMC2729606

[BIO013128C12] Higuchi M., Pischke M. S., Mahonen A. P., Miyawaki K., Hashimoto Y., Seki M., Kobayashi M., Shinozaki K., Kato T., Tabata S. et al. (2004). In planta functions of the Arabidopsis cytokinin receptor family. *Proc. Natl. Acad. Sci. USA* 101, 8821-8826. 10.1073/pnas.0402887101 15166290 PMC423279

[BIO013128C13] Horiguchi G., Gonzalez N., Beemster G. T. S., Inzé D. and Tsukaya H. (2009). Impact of segmental chromosomal duplications on leaf size in the grandifolia-D mutants of Arabidopsis thaliana. *Plant J.* 60, 122-133. 10.1111/j.1365-313X.2009.03940.x 19508432

[BIO013128C14] Karimi M., Inzé D. and Depicker A. (2002). GATEWAY™ vectors for Agrobacterium-mediated plant transformation. *Trends Plant Sci.* 7, 193-195. 10.1016/S1360-1385(02)02251-3 11992820

[BIO013128C15] Karlberg A., Bako L. and Bhalerao R. P. (2011). Short day-mediated cessation of growth requires the downregulation of AINTEGUMENTALIKE1 transcription factor in hybrid aspen. *PLoS Genet.* 7, e1002361 10.1371/journal.pgen.1002361 22072988 PMC3207903

[BIO013128C16] Kim S., Soltis P. S., Wall K. and Soltis D. E. (2006). Phylogeny and domain evolution in the APETALA2-like gene family. *Mol. Biol. Evol.* 23, 107-120. 10.1093/molbev/msj014 16151182

[BIO013128C17] Livak K. J. and Schmittgen T. D. (2001). Analysis of relative gene expression data using real-time quantitative PCR and the 2(-Delta Delta C(T)) Method. *Methods* 25, 402-408. 10.1006/meth.2001.1262 11846609

[BIO013128C18] Mahonen A. P., Bonke M., Kauppinen L., Riikonen M., Benfey P. N. and Helariutta Y. (2000). A novel two-component hybrid molecule regulates vascular morphogenesis of the Arabidopsis root. *Genes Dev.* 14, 2938-2943. 10.1101/gad.189200 11114883 PMC317089

[BIO013128C19] Mahonen A. P., Bishopp A., Higuchi M., Nieminen K. M., Kinoshita K., Tormakangas K., Ikeda Y., Oka A., Kakimoto T. and Helariutta Y. (2006). Cytokinin signaling and its inhibitor AHP6 regulate cell fate during vascular development. *Science* 311, 94-98. 10.1126/science.1118875 16400151

[BIO013128C20] Matsumoto-Kitano M., Kusumoto T., Tarkowski P., Kinoshita-Tsujimura K., Vaclavikova K., Miyawaki K. and Kakimoto T. (2008). Cytokinins are central regulators of cambial activity. *Proc. Natl. Acad. Sci. USA* 105, 20027-20031. 10.1073/pnas.0805619105 19074290 PMC2605004

[BIO013128C21] Menges M. and Murray J. A. H. (2002). Synchronous Arabidopsis suspension cultures for analysis of cell-cycle gene activity. *Plant J.* 30, 203-212. 10.1046/j.1365-313X.2002.01274.x 12000456

[BIO013128C22] Menges M., Samland A. K., Planchais S. and Murray J. A. H. (2006). The D-type cyclin CYCD3;1 is limiting for the G1-to-S-phase transition in Arabidopsis. *Plant Cell* 18, 893-906. 10.1105/tpc.105.039636 16517759 PMC1425856

[BIO013128C23] Menges M., Pavesi G., Morandini P., Bogre L. and Murray J. A. H. (2007). Genomic organization and evolutionary conservation of plant D-type cyclins. *Plant Physiol.* 145, 1558-1576. 10.1104/pp.107.104901 17951462 PMC2151690

[BIO013128C24] Miyashima S., Sebastian J., Lee J.-Y. and Helariutta Y. (2013). Stem cell function during plant vascular development. *EMBO J.* 32, 178-193. 10.1038/emboj.2012.301 23169537 PMC3553377

[BIO013128C25] Miyawaki K., Tarkowski P., Matsumoto-Kitano M., Kato T., Sato S., Tarkowska D., Tabata S., Sandberg G. and Kakimoto T. (2006). Roles of Arabidopsis ATP/ADP isopentenyltransferases and tRNA isopentenyltransferases in cytokinin biosynthesis. *Proc. Natl. Acad. Sci. USA* 103, 16598-16603. 10.1073/pnas.0603522103 17062755 PMC1637627

[BIO013128C26] Mizukami Y. and Fischer R. L. (2000). Plant organ size control: AINTEGUMENTA regulates growth and cell numbers during organogenesis. *Proc. Natl. Acad. Sci. USA* 97, 942-947. 10.1073/pnas.97.2.942 10639184 PMC15435

[BIO013128C27] Morgan D. O. (1997). Cyclin-dependent kinases: engines, clocks, and microprocessors. *Annu. Rev. Cell Dev. Biol.* 13, 261-291. 10.1146/annurev.cellbio.13.1.261 9442875

[BIO013128C28] Nieminen K. M., Kauppinen L. and Helariutta Y. (2004). A weed for wood? Arabidopsis as a genetic model for xylem development. *Plant Physiol.* 135, 653-659. 10.1104/pp.104.040212 15208411 PMC514101

[BIO013128C29] Nieminen K., Immanen J., Laxell M., Kauppinen L., Tarkowski P., Dolezal K., Tahtiharju S., Elo A., Decourteix M., Ljung K. et al. (2008). Cytokinin signaling regulates cambial development in poplar. *Proc. Natl. Acad. Sci. USA* 105, 20032-20037. 10.1073/pnas.0805617106 19064928 PMC2604918

[BIO013128C30] Nieuwland J., Maughan S., Dewitte W., Scofield S., Sanz L. and Murray J. A. H. (2009). The D-type cyclin CYCD4;1 modulates lateral root density in Arabidopsis by affecting the basal meristem region. *Proc. Natl. Acad. Sci. USA* 106, 22528-22533. 10.1073/pnas.0906354106 20018777 PMC2794031

[BIO013128C31] Nishimura C., Ohashi Y., Sato S., Kato T., Tabata S. and Ueguchi C. (2004). Histidine kinase homologs that act as cytokinin receptors possess overlapping functions in the regulation of shoot and root growth in Arabidopsis. *Plant Cell* 16, 1365-1377. 10.1105/tpc.021477 15155880 PMC490032

[BIO013128C32] Nole-Wilson S., Tranby T. L. and Krizek B. A. (2005). AINTEGUMENTA-like (AIL) genes are expressed in young tissues and may specify meristematic or division-competent states. *Plant Mol. Biol.* 57, 613-628. 10.1007/s11103-005-0955-6 15988559

[BIO013128C33] Parinov S., Sevugan M., Ye D., Yang W.-C., Kumaran M. and Sundaresan V. (1999). Analysis of flanking sequences from dissociation insertion lines: a database for reverse genetics in Arabidopsis. *Plant Cell* 11, 2263-2270. 10.1105/tpc.11.12.2263 10590156 PMC144131

[BIO013128C34] Randall R. S., Sornay E., Dewitte W. and Murray J. A. H. (2015). AINTEGUMENTA and the D-type cyclin CYCD3;1 independently contribute to petal size control in Arabidopsis: evidence for organ size compensation being an emergent rather than a determined property. *J. Exp. Bot.* 66, 3991-4000. 10.1093/jxb/erv200 25948704 PMC4473993

[BIO013128C35] Renaudin J.-P., Doonan J. H., Freeman D., Hashimoto J., Hirt H., Inze D., Jacobs T., Kouchi H., Rouze P., Sauter M. et al. (1996). Plant cyclins: a unified nomenclature for plant A-, B- and D-type cyclins based on sequence organization. *Plant Mol. Biol.* 32, 1003-1018. 10.1007/BF00041384 9002599

[BIO013128C36] Riou-Khamlichi C., Huntley R., Jacqmard A. and Murray J. A. H. (1999). Cytokinin activation of Arabidopsis cell division through a D-type cyclin. *Science* 283, 1541-1544. 10.1126/science.283.5407.1541 10066178

[BIO013128C37] Schrader J., Nilsson J., Mellerowicz E., Berglund A., Nilsson P., Hertzberg M. and Sandberg G. (2004). A high-resolution transcript profile across the wood-forming meristem of poplar identifies potential regulators of cambial stem cell identity. *Plant Cell* 16, 2278-2292. 10.1105/tpc.104.024190 15316113 PMC520933

[BIO013128C38] Schruff M. C., Spielman M., Tiwari S., Adams S., Fenby N. and Scott R. J. (2006). The AUXIN RESPONSE FACTOR 2 gene of Arabidopsis links auxin signalling, cell division, and the size of seeds and other organs. *Development* 133, 251-261. 10.1242/dev.02194 16339187

[BIO013128C39] Spicer R. and Groover A. (2010). Evolution of development of vascular cambia and secondary growth. *New Phytol.* 186, 577-592. 10.1111/j.1469-8137.2010.03236.x 20522166

[BIO013128C40] Werner T., Motyka V., Strnad M. and Schmülling T. (2001). Regulation of plant growth by cytokinin. *Proc. Natl. Acad. Sci. USA* 98, 10487-10492. 10.1073/pnas.171304098 11504909 PMC56987

[BIO013128C41] Wu B., Li Y.-H., Wu J.-Y., Chen Q.-Z., Huang X., Chen Y.-F. and Huang X.-L. (2011). Over-expression of mango (Mangifera indica L.) MiARF2 inhibits root and hypocotyl growth of Arabidopsis. *Mol. Biol. Rep.* 38, 3189-3194. 10.1007/s11033-010-9990-8 20182802

[BIO013128C42] Zhang J., Gao G., Chen J.-J., Taylor G., Cui K.-M. and He X.-Q. (2011). Molecular features of secondary vascular tissue regeneration after bark girdling in Populus. *New Phytol.* 192, 869-884. 10.1111/j.1469-8137.2011.03855.x 21883236

